# Angiogenesis related genes based prognostic model of glioma patients developed by multi-omics approach

**DOI:** 10.1007/s12672-024-01126-6

**Published:** 2024-07-20

**Authors:** Zhimin Liu, Hongjun Fan, XuKai Liu, Chao liu

**Affiliations:** Department of Neurosurgery, Central Hospital of Zhuzhou, Zhuzhou, Hunan China

**Keywords:** Glioma, Angiogenesis, Prognosis, Tumor microenvironment, Machine learning, Heterogeneity

## Abstract

**Introduction:**

Glioma, particularly glioblastoma (GBM), is a highly malignant brain tumor with poor prognosis despite current therapeutic approaches. The tumor microenvironment (TME), plays a crucial role in glioma progression by promoting invasion and drug resistance. Angiogenesis, the formation of new blood vessels, is a tightly regulated process involving endothelial cell activation, proliferation, and migration. In cancer, angiogenesis becomes dysregulated, leading to excessive blood vessel formation.

**Methods:**

We enrolled bulk data of TCGA-LGG/GBM, CGGA-693, and CGGA-325 cohorts, scRNA data of GSE162631, GSE84465, and GSE138794 cohorts. Identification of malignant cells was conducted by “copycat” R package. The “AUCell” R package scored the activity of target gene set of each single cell. Consensus clustering was applied using the “ConsensusClusterPlus” R package, while tumor-infiltrating immune cells were determined using “IOBR” R package. To construct a prognostic model, we used LASSO and multiCOX algorithms based on the expression levels of the 15 hub genes, the efficacy of which was verified by KM and ROC analysis.

**Results:**

We identified 4 different malignant cell subclusters in glioma and disclosed their distinct gene expression patterns and interactions within TME. We identified differentially expressed immune-related genes (DE-ARGs) in glioma and found 15 genes that were specifically expressed in the malignant glioma cell populations. Glioma cells with higher expression of these DE-ARGs were associated with gliogenesis, glial cell development, and vasculature development. We found that tumor-infiltrating monocytes were the main interacting cell type within glioma TME. Using the expression patterns of the 15 screened DE-ARGs, we categorized glioma samples into 2 molecular clusters with distinct immune features, suggesting a possible relationship between angiogenesis and immune activation and recruitment. We constructed a prognostic model based on the expression levels of the 15 DE-ARGs and evaluated its predictive ability for glioma patient outcomes, which displayed exceedingly high efficacy.

**Conclusion:**

We characterized different malignant cell subclusters in glioma and investigate their gene expression patterns and interactions within TME. We constructed a prognostic model based on the expression levels of the 15 DE-ARGs and evaluated its predictive ability for glioma patient outcomes, which displayed exceedingly high efficacy.

## Introduction

Glioma is the most common form of primary malignant brain tumor with significant heterogeneity [1]. Although various therapeutic approaches, such as surgery, radiotherapy, and chemotherapy, have been utilized, the overall prognosis for glioma patients remains poor [2]. Among glioma, glioblastoma (GBM) is the most malignant subtype, lacking promising progress in its treatment. The 5 year survival rate for GBM is near to 5% [3]. The tumor microenvironment (TME), consisting of complex cellular and non-cellular components surrounding the tumor, plays a crucial role in glioma progression [4]. In particular, tumor-associated macrophages (TAMs) in the GBM microenvironment have been found to promote invasion and drug resistance by altering the tumor immune microenvironment [5]. A deeper insight into the molecular heterogeneity underlying glioma might provide great advantage for customized treatment.

Angiogenesis is a tightly regulated process in which new blood vessels form from existing capillaries. This process involves the degradation of the basement membrane and the activation, proliferation, and migration of endothelial cells (ECs) [6]. Under physiological conditions, ECs are mostly quiescent, with a low frequency of mitosis [7]. Angiogenesis occurs in various physiological processes such as embryonic development, tissue repair, and organ regeneration, but is strictly controlled, localized, and transient. However, in certain diseases, including immune diseases and various cancers, angiogenesis becomes dysregulated and excessive [8]. This is characterized by the over-expression of pro-angiogenic factors and the inactivation of anti-angiogenic factors. Dysregulated angiogenesis contributes to cancer progression and is considered a promising therapeutic target. Through multi-omics analysis, Su et al*.* proposed novel prognosis models for prostate cancer patients, which significantly improved the accuracy of clinical outcomes prediction [9, 10]. However, in glioma, evidences regarding the role of angiogenesis in molecular identification and prognosis of glioma patients remains vague.

In this study, we aimed to identify different malignant cell subclusters in glioma and investigate their gene expression patterns and interactions within TME. We analyzed scRNA-seq data from glioma samples and identified 4 distinct subclusters of malignant glioma cells. Differential expression of certain genes, such as CHI3L1, MEG3, FOS, and CCL4L2, were observed between these subclusters, along with differential enrichment of crucial pathways, including cell communication, cell differentiation, immune response, and inflammatory response, indicating significant intratumor heterogeneity within glioma samples. We intersected the identified differentially expressed genes (DEGs) and angiogenesis related genes (ARGs), generating a total of 15 screened DE-ARGs. We constructed a prognostic model based on the expression levels of the 15 DE-ARGs and evaluated its predictive ability for glioma patient outcomes, which displayed exceedingly high efficacy. Hopefully, our study provides insights into the molecular subtypes of glioma and their implications for glioma patient prognosis.

## Methods

### Data acqusition and preprocessing

The mRNA expression of the TCGA-LGG/GBM cohort was obtained from the TCGA database (https://portal.gdc.cancer.gov/). CGGA-693 and CGGA-325 datasets were downloaded from the CGGA database (http://www.cgga.org.cn/). Single-cell dataset of GSE162631, GSE84465, and GSE138794 was downloaded from the GEO database (www.ncbi.nlm.nih.gov/geo/). The data used in this study are from public databases, and no additional ethical approval is required, and our analysis process complies with relevant regulations.

### ScRNA-seq procurement and analysis

After quality control filtration, harmony data set was used for de-batch integration to obtain 78738 glioma single cells. Under the resolution of 0.2, copolymerization was performed to 12 cell groups, and 7 major cell categories were obtained by SingleR annotation. A total of 24,981 single cells were extracted from 7 cell types and further divided into 10 glial cell groups under the resolution of 0.2. Copykat recognizes malignant glioma cells in the cell population; a total of 14,074 single cells from the malignant glioma cell population were further divided into 4 groups, and four malignant glioma cell populations were copolymerized under the resolution of 0.2. To identify up-regulated and down-regulated genes in each malignant glioma cell population based on RunDEtest function; featureHeatmap function identified highly expressed genes in each malignant glioma subgroup. TF and CSPA specific to each cell subgroup, and GOBP functional annotation was performed for each malignant glioma subgroup. RunGSEA function recognizes the up-regulated Gene Set Enrichment Analysis (GSEA) pathway for each malignant glioma subgroup.

### Identification of target genes associated with angiogenesis and glioma malignant phenotypes

The FindAllMarkers function in the “Seurat” R package (v5.0.0) was used to identify specific markers of four malignant glioma cell populations (14,074 cells), and then intersected with 4730 ARGs to obtain a total of 15 genes. The correlation analysis of 15 common genes was performed using 704 tumor tissues in the TCGA-LGG / GBM dataset, and the expression of 15 common genes at the single cell level was displayed using the integrated 78,738 glioma single cell dataset.

### AUCell based scoring of glioma single cells using the screened target genes

The “AUCell” R package(v1.24.0) scored the activity of the gene set (15 differentially expressed ARGs) of a single cell, and divided the 24,981 glioma malignant cells into high-/low-scores according to the threshold, and then GSEA enrichment analysis was performed to find up-regulated pathways or functions in the high-score cell population.

### Development trajectory analysis and cell chat analysis of malignant glioma cells

In the 14,074 malignant glioma single cell data set, the marker genes up-regulated in each cell subgroup were identified, and the expression of 15 DE-ARGs at the single cell level was displayed. Then monocle2 was used to display the development trajectory of malignant glioma, and it was inferred that tumor stem cell subsets may be obtained. Show the expression changes of these 15 differentially expressed ARGs over time. The "cellchat" R package was used to identify LR pairs in malignant glioma cells.

### Consensus clustering and TME evaluation

Consensus clustering was applied to stratify glioma into distinct molecular clusters based on the expression of 15 screened hub genes using the “ConsensusClusterPlus” R package. The clustering was conducted with specific parameters and repetitions, and the optimal number of clusters was determined using consensus score matrix, PAC scores, and CDF curves. The infiltration abundances of tumor-infiltrating immune cells were determined using various TME-decoding algorithms wrapped in the “IOBR” R package.

### Prognosis model construction and nomogram model establishment

Based on 15 hub genes, a prognostic model was constructed using LASSO and multiCOX. The risk score is obtained by multiplying the amount of gene expression by the corresponding coefficient and then adding it. The KM curve and ROC curve were used to evaluate the predictive ability of the model for prognosis in each data set. UniCox demonstrates the prognostic value of each gene. Finally, a nomogram including riskScore and age was constructed.

### Statistical analysis

In this study, R 4.1.3 software was used for all data processing, statistical analysis, and data visualization. To determine the correlation between two continuous variables, the Pearson correlation coefficient was utilized. Additionally, the association between survival outcomes and predictor variables was investigated using both Cox regression analysis and Kaplan–Meier analysis, which were performed using the “survival” package. We used statistical significance P-value less than 0.05 as the criterion to judge statistical significance.

## Results

### The identification of 4 glioma malignant cell subclusters

**Fig. 1:**
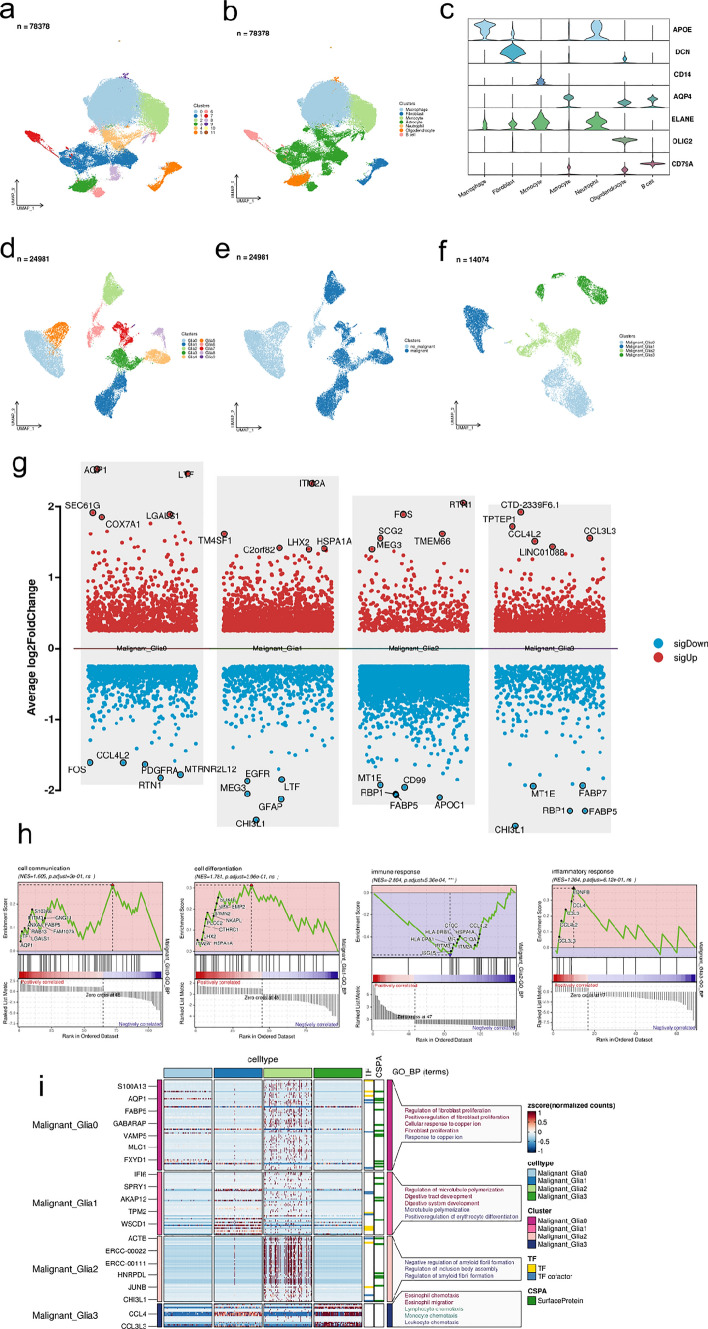
Cell type annotation and functional analysis of the glioma at single cell resolution. **a** UMAP of 12 divided cell clusters under the resolution of 0.2. **b** UMAP of 7 cell clusters annotated by “SingleR” R package. **c** Violin plots of the expression of specific marker genes in each cell cluster. **d** UMAP of 10 glia cell clusters under the resolution of 0.2. **e** UMAP visualization of malignant glioma cells identified by “copycat” R package. **f** UMAP of 4 malignant glia cell clusters under the resolution of 0.2. **g** Significantly up-regulated and down-regulated genes of each malignant glioma cell cluster. **h** Significantly activated pathway in each malignant glia cell cluster. **i** Heatmap showing the specific markers and ighly enriched pathways of each malignant glioma cell cluster

After conducting quality control filtration, we utilized the harmony dataset for de-batch integration, resulting in a total of 78,738 glioma single cells. These cells were grouped into 12 cell groups using copolymerization with a resolution of 0.2 (Fig. 1a). SingleR annotation was then employed to identify 7 major cell categories (Fig. 1b, c). From these categories, we extracted 24,981 single cells representing 7 different cell types. These cells were further subdivided into 10 glioma malignant cell subgroups at a resolution of 0.2 (Fig. 1d). The malignant cells accounted for majority of the annotated glioma cells (Fig. 1e). Additionally, using Copykat, a total of 14,074 single cells from the malignant glioma cell population were assigned to 4 subgroups, and these four malignant glioma cell populations were copolymerized at a resolution of 0.2 (Fig. 1f). We found CHI3L1 significantly down-regulated in the malignant glioma subclusters 1 and 3 (Fig. 1g). Distinct differential expression of MEG3 was observed in the malignant glia clusters 1 and 2. Similar pattern was observed in the expression level of FOS in the malignant glia clusters 0, 2 and CCL4L2 between the subcluster 0, 3, suggesting these genes might be vitally implicated in the progression of certain type of glioma malignant cells. We observed significant enrichment of cell communication, cell differentiation, immune response and inflammatory response in subcluster 0 to 3 of malignant glioma cells, sustaining prominent intratumor heterogeneity within the glioma samples (Fig. 1h). The subcluster 0, which was characterized with enhanced cell communication capability, was shown to be highly associated with regulation of fibroblast proliferation process, suggesting subcluster 0 might play a pivotal role in regulating CAF differentiation (Fig. 1i). On the other hand, subcluster 3 could be a universal lymphocyte recruiter in glioma TME.

### Identification of differentially expressed ARGs in glioma

**Fig. 2:**
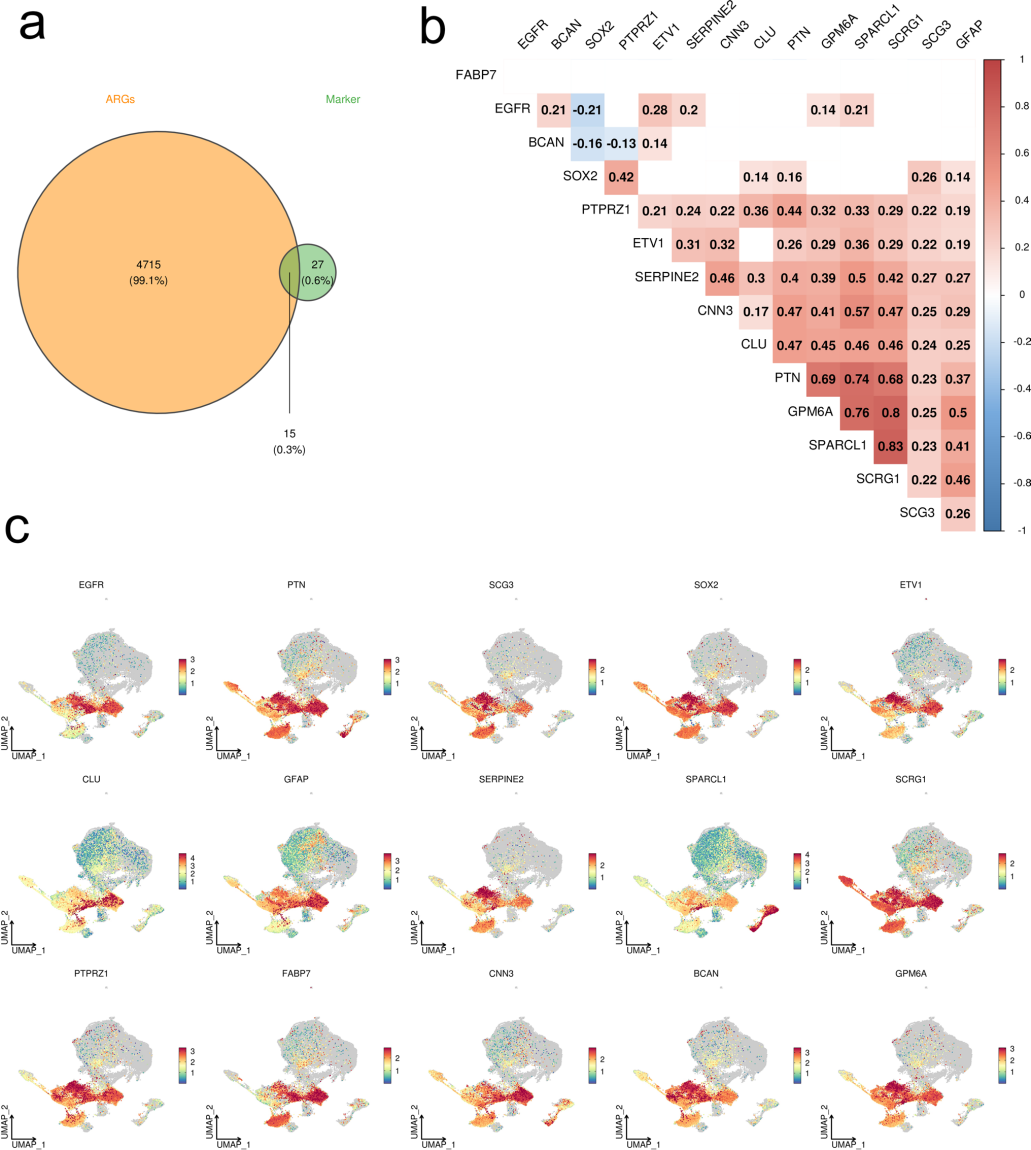
Identification of differentially expressed ARGs based on scRNA-seq. **a** Venn diagram showing the intersection and identification of DE-ARGs between differentially expressed cell marker genes and ARGs. **b** Correlation analysis of 15 DE-ARGs, displayed in the form of heatmap. **c** UMAP plots showing the expression levels of 15 DE-ARGs in different cell clusters

**Fig. 3:**
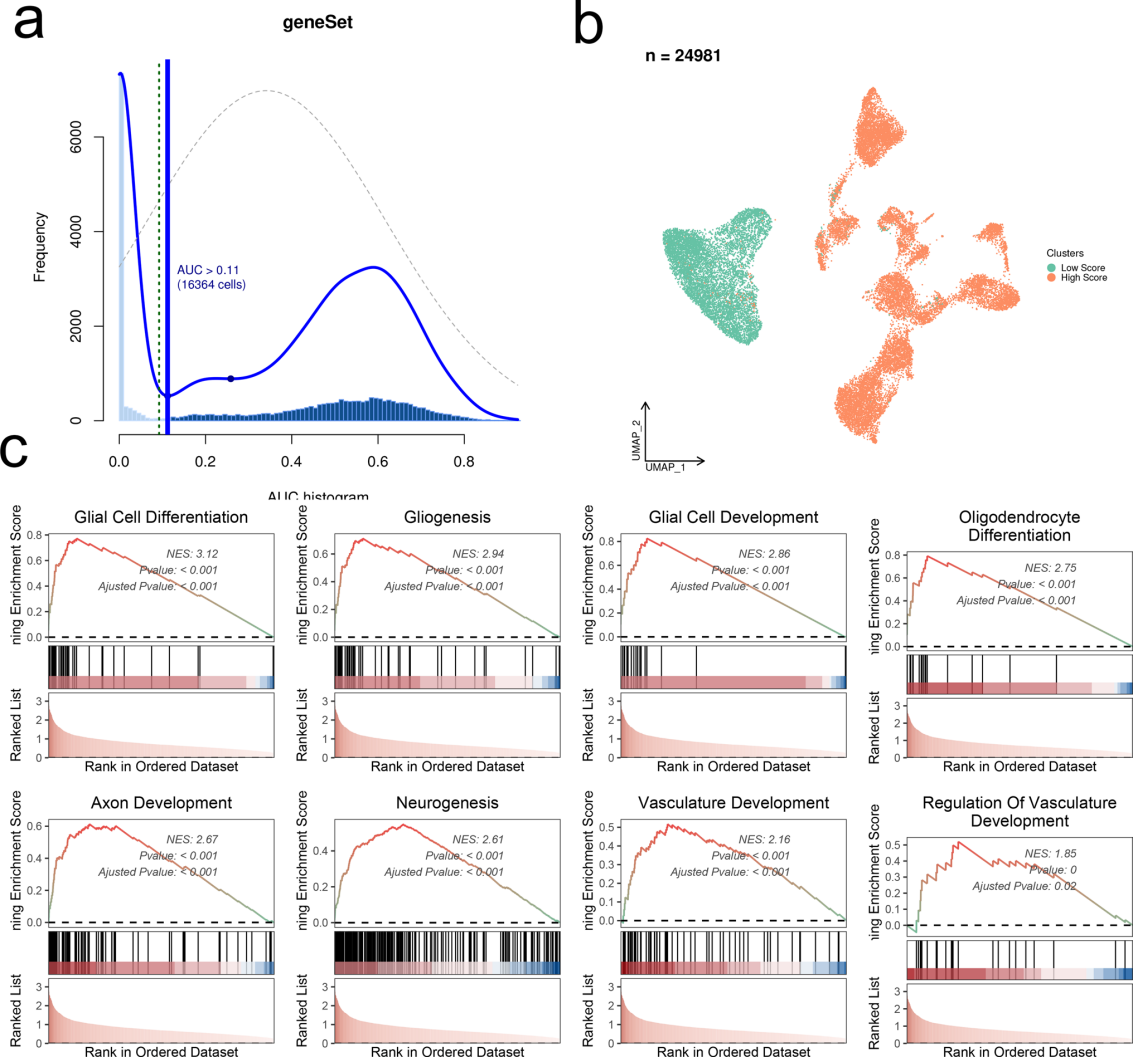
Scoring of 15 DE-ARGs. **a** AUC histogram of 15 DE-ARGs. **b** UMAP plot based on ARG score of each cell. **c** GSEA analysis of the DEGs in cells with high-ARG scores

**Fig. 4:**
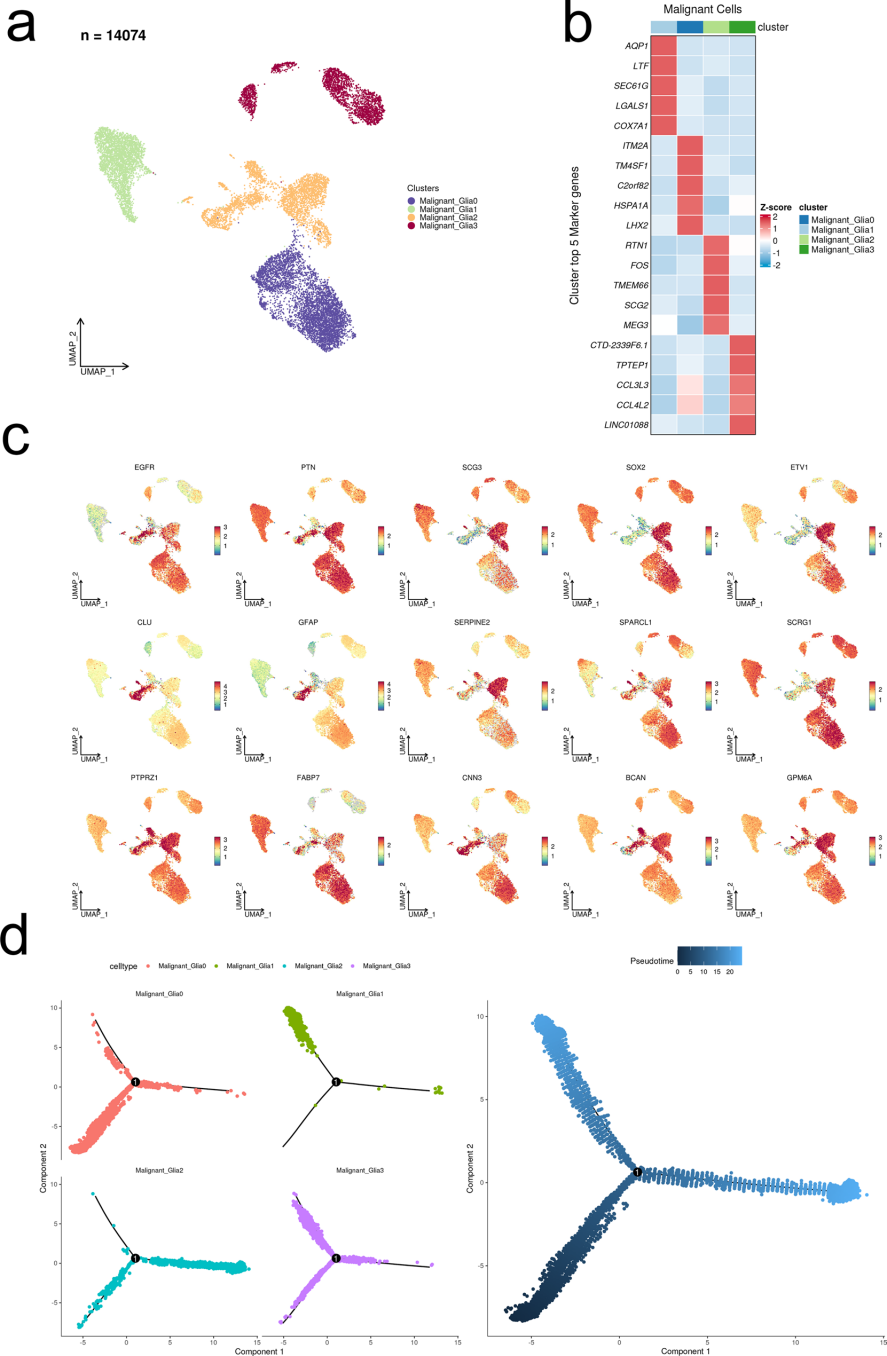
Subdivision of malignant glioma cells and trajectory analysis. **a** UMAP plot of 4 malignant glia cell subpopulations in glioma. **b** Heatmap of the expression of cell marker genes in 4 malignant glioma cell subpopulations. **c** UMAP plots of 15 DE-ARGs in 4 cell subpopulations. **d** Differentiation trajectory of glia cells, colored for cell types (left), and pseudotime (right)

**Fig. 5:**
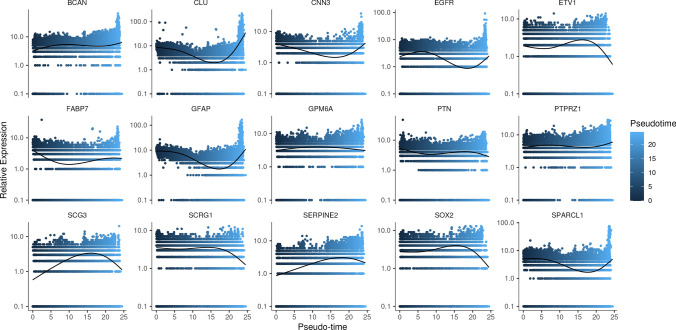
Relative expression profiles of 15 DE-ARGs during the differentiation pseudo-time trajectory

The FindAllMarkers function was used to identify specific markers (42 genes) of four malignant glioma cell populations, and then intersected with 4730 ARGs to obtain a total of 15 genes (Fig. 2a). Correlation analysis displayed intense correlative relationship between SCRG1 and SPARCL1, GPM6A (Fig. 2b). CLU, CNN3, PTPRZ1 and GFAP were found to be highly expressed in the astrocytes among the 7 clusters (Fig. 2c). We applied the “AUCell” R package to score the activity of the differentially expressed ARGs gene set of each single cell, which generated a total of 16,364 cells with AUC > 0.11 (Fig. 3a). As shown in the Fig. 3b, the glioma malignant cells with relatively high AUC value accounted for the majority of the whole population (Fig. 3b). We divided the whole glioma malignant cell population into high- and low-score subgroups according to the mentioned threshold, to which we applied GSEA enrichment analysis for investigation of the up-regulated pathways in the high-score glioma cell population. Glioma cells with higher differentially expressed ARGs gene set scores were featured with gliogenesis, glial cell development, vasculature development, which was consistent with the expectation (Fig. 3c). We displayed the clustering status of the malignant glioma cells in the form of UMAP (Fig. 4a). The corresponding marker genes expression pattern was shown in the Fig. 4b. The detailed expression of each marker gene was displayed. EGFR and PTN were mainly expressed in the subcluster 0, while chemokines, including CCL3L3 and CCL4L2 were mainly enriched in the subcluster 3 (Fig. 4c). Differentiation trajectory of targeted glioma cells was delineated, revealing malignant glioma cell subcluster 0, 1, and 2 as the terminal differentiation state (Fig. 4d), while subcluster 3 was identified as the intermediate state of glioma malignant cells. By the calculated pseudotime value, we managed to identify the malignant glia subcluster 1 and 2 as the 2 final stages originating from the subcluster 0. We mapped the expression of 15 screened DE-ARGs onto the malignant cells aligned by the pseudotime value (Fig. 5). BCAN and SERPINE2 were gradually elevating following the differentiation, while SCRG1 displayed a diminishing pattern. SOX2, a well-established marker of glioma stemness, was found to be slightly elevated in the middle stage and significantly down-regulated in the terminal stages.

### Cell communication landscaping in the glioma at single cell resolution

**Fig. 6:**
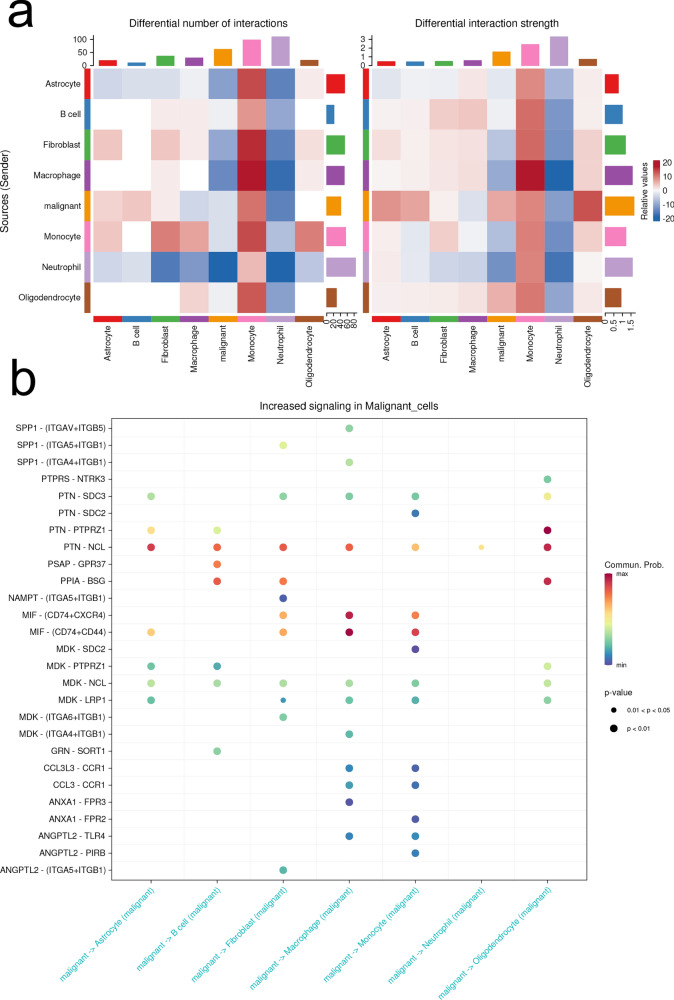
Cell–cell communication analysis. **a** Cell–cell communication displaying interaction numbers (left) and interaction strength (right). Blue blocks indicate downregulated interaction, whereas red blocks represent upregulated communication in glioma. **b** Upregulated receptor-ligand interaction networks from malignant glia cells to other cell subsets. The size of dots represents P-value. Red dot indicates a higher communication probability and blue dot represents a lower communication probability

We found the tumor-infiltrating monocytes as the main interacting cell type in the TME. On the other hand, neutrophil was identified with lowest interaction intensity (Fig. 6a). The highest interaction was found between the intratumor monocytes and macrophages, fibroblast, suggesting an auto-secretory phenotype of the macrophages and para-secretory pattern between CAFs and CAMs, suggesting a heated interaction in the glioma TME. Next, we further investigated the signal transduction originating from the malignant glioma cells (Fig. 6b). The highest LR interaction was found between malignant cells and CAMs with highly activated MIF signaling (CD74 + CXCR4, CD74 + CD44) and PTN-NCL signaling. The interaction between malignant glioma cells and CAFs was mainly conducted through PTN-NCL and PPIA-BSG signaling pathways. Little interaction was found between the malignant cells and neutrophil, suggesting the intratumor neutrophil with no signaling function.

### Identification of DEGs based on transcriptomics

**Fig. 7:**
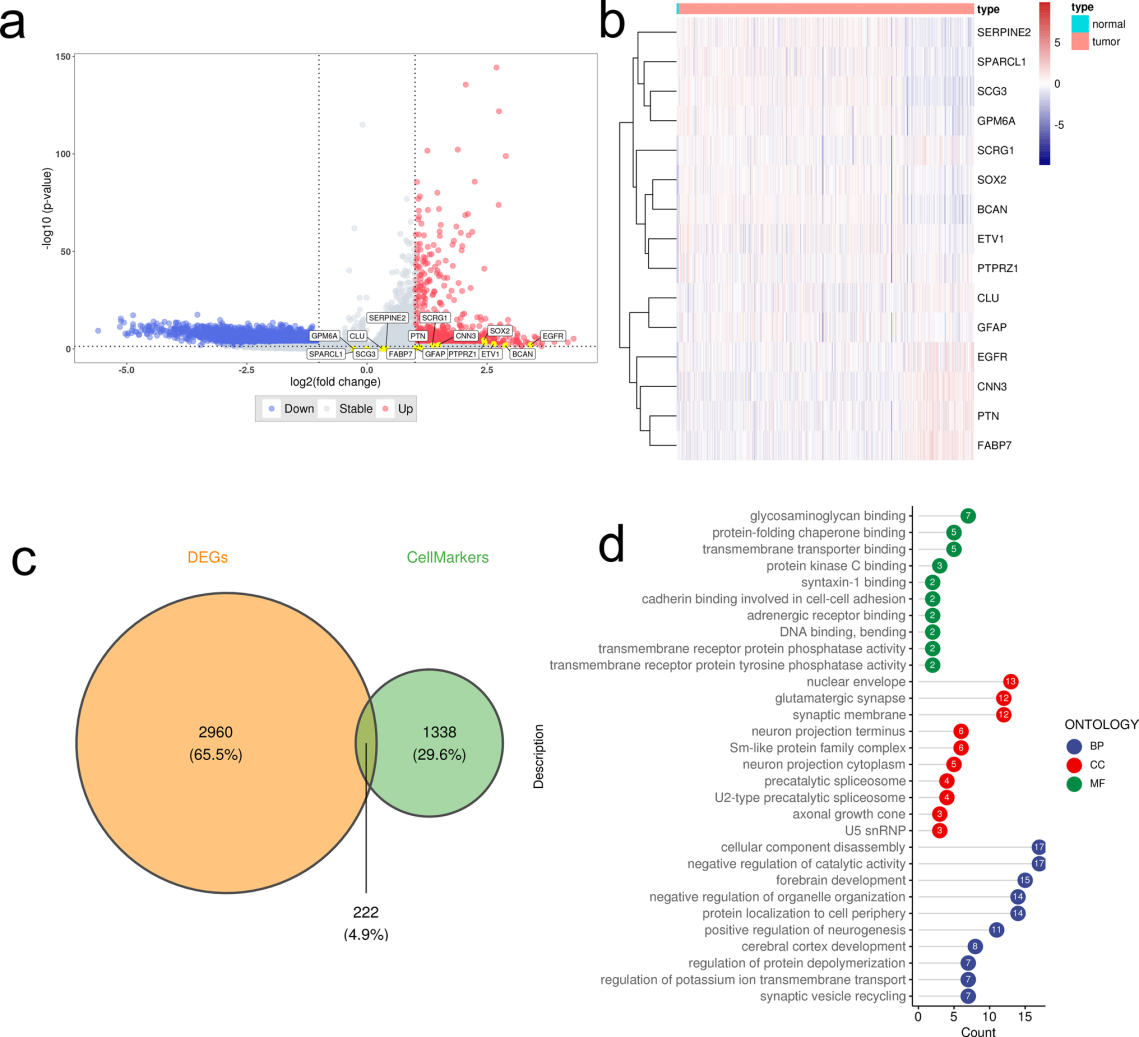
Identification of DEGs based on transcriptomics. **a** Volcanic map for DEGs between normal and tumor groups based on transcriptome sequencing results. Blue dots represent downregulated genes in glioma, and red dots indicate upregulated genes in glioma. **b** Heatmap for DEGs between normal and tumor groups based on transcriptome sequencing results. **c** Venn diagram indicates 222 overlapping DEGs between screened cell marker genes and bulk transcriptome. **d** GO analysis of 222 DEGs

We compared the DEGs between normal and tumor groups based on transcriptome sequencing results (Fig. 7a). A consistent result of distinguishable DEGs could be observed between the tumorous tissue and para-tumor tissue. EGFR, BCAN, SOX2, PTN and CCN3 were found to be highly elevated in the glioma tumorous tissue with prominent fold change value, while other DE-ARGs, such as CLU, SCG3, showed no distinct expression alteration (Fig. 7b). The intersection with DEGs obtained a total of 222 genes (Fig. 7c), which were subsequently subjected to GO functional analysis using clusterprofiler (Fig. 7d). We observed a high enrichment of the negative regulation of catalytic activity and positive regulation of neurogenesis, suggesting a metabolic shift towards cell proliferation.

### Molecular subcluster of the glioma based on 15 screened DE-ARGs

**Fig. 8:**
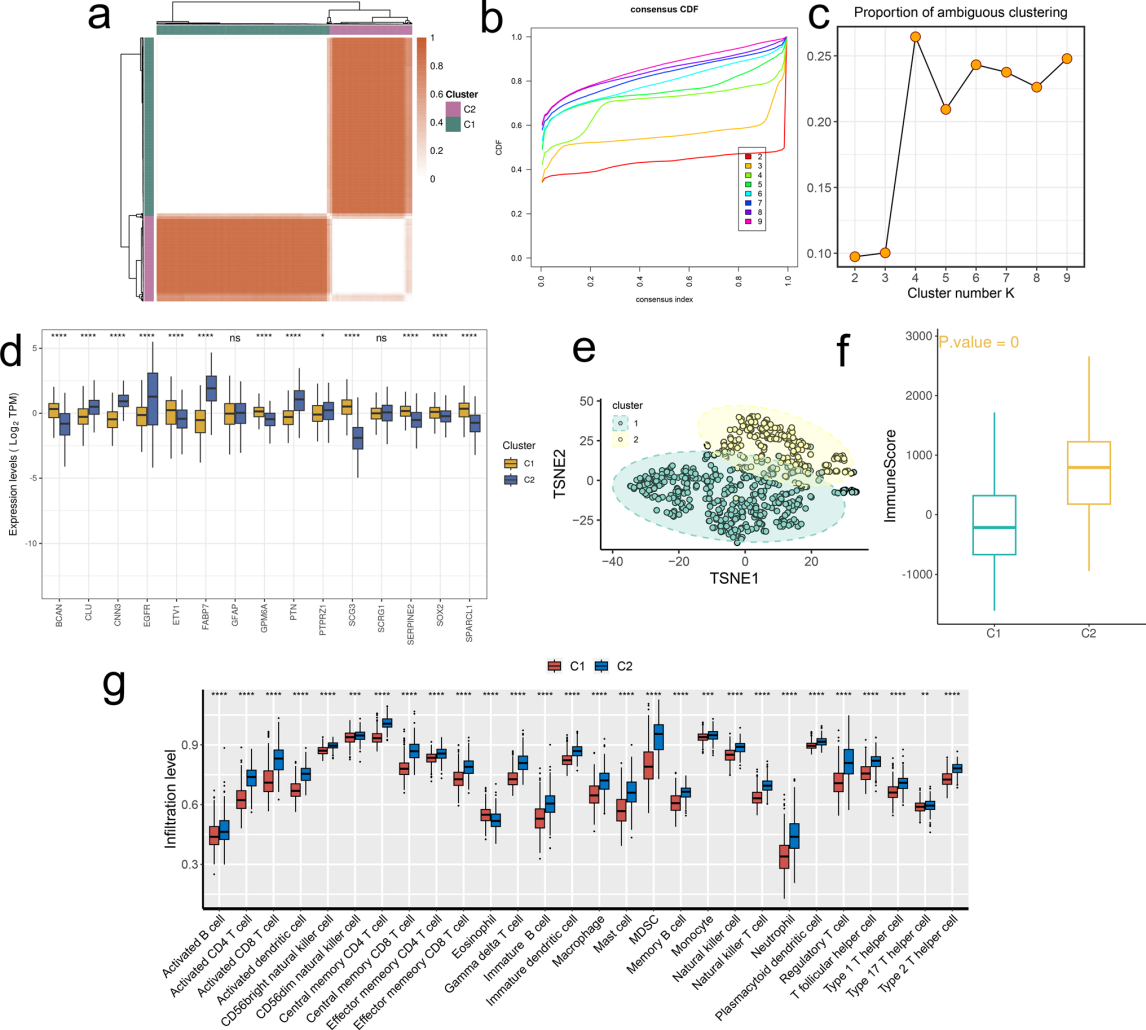
Consensus clustering analysis. **a** Heatmap of clustering at consensus k = 2. **b** Cumulative distribution function (CDF) curves of different consensus k-values. **c** The proportion of ambiguous clustering (PAC) score, a low value of PAC implies a flat middle segment, allowing conjecture of the optimal k (k = 2) by the lowest PAC. **d** Difference of 15 DE-ARGs expression levels between 2 clusters. **e** Principal component analysis of 2 clusters. **f** Comparison of immune score between 2 clusters. **g** Difference of immune infiltration score between 2 clusters calculated by ssGSEA. *P < 0.05, **P < 0.01, ***P < 0.001, ****P < 0.0001

A consensus clustering approach was employed to categorize glioma samples into distinct molecular clusters based on the expression patterns of 15 screened DE-ARGs. We chose k = 2 for subsequent clustering (Fig. 8a), which was supported by the CDF curve and proportion of ambiguous clustering analysis results (Fig. 8b, c). Further expression pattern analysis of the 15 screened DE-ARGs disclosed CLU, CNN3, EGFR, PTN and FABP7 highly elevated in the C2 cluster (Fig. 8d). The PCA analysis showed a distinguishable separation of C1 and C2, sustaining the accuracy of our clustering strategy (Fig. 8e). We evaluated the immune status in 2 subclusters of glioma cells, which showed that the immune score of TME of C2 were markedly higher (Fig. 8f). Consistently, activated CD8 + , CD4 + , B cells and NKT cells were found to be of greater abundance in the C2 cluster (Fig. 8g), suggesting an overactive TME in the C2.

### Prognostic model construction and nomogram establishment

**Fig. 9:**
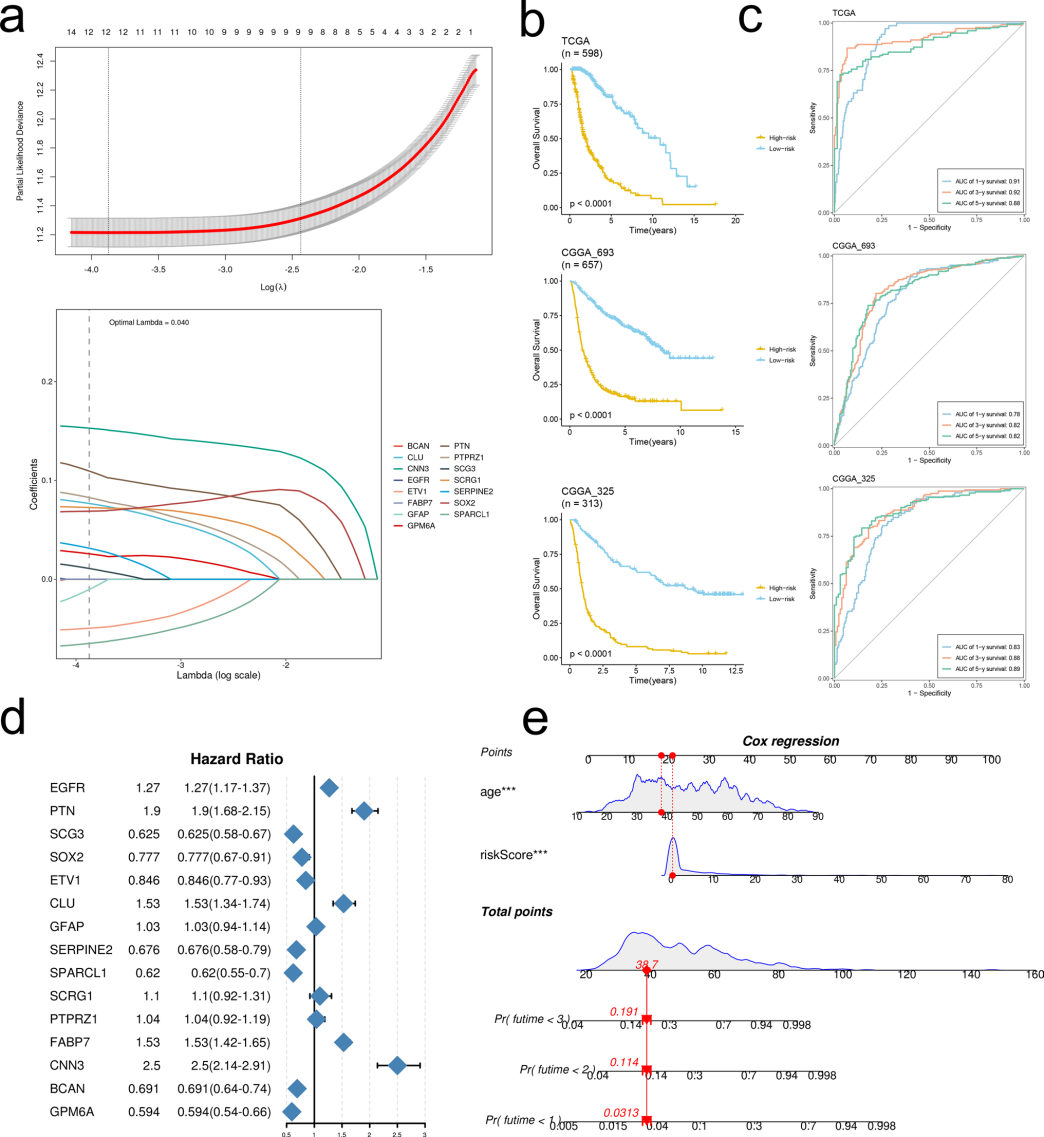
Construction and validation of the prognostic risk model. **a** The selection of prognostic DE-ARGs based on the optimal parameter λ that was obtained in the LASSO regression analysis. **b** K-M curves displayed survival outcomes of patients in two risk groups from the three cohorts. **c** Time-dependent ROC curves were drawn to assess survival rate at 1 year, 3 year, and 5 year in the three cohort. **d** Univariate cox regression analysis of 15 DE-ARGs in TCGA cohort. **e** The nomogram was constructed with different factors, including risk model (risk score) and age

A prognostic model was constructed based on the expression levels of 15 DE-ARGs using LASSO (Fig. 9a). The risk factor formula was: PTN * 0.148484225 + SCG3 * (− 0.030255705) + SOX2 * (− 0.067584106) + ETV1 * (− 0.056611966) + CLU * 0.093226743 + GFAP * (− 0.064655375) + SERPINE2 * 0.053830624 + SPARCL1 * (− 0.075747892) + SCRG1 * 0.077039162 + PTPRZ1 * 0.104935377 + CNN3 * 0.162388743 + GPM6A * (− 0.037791524). K-M curves displayed survival outcomes of glioma patients in two risk groups (Fig. 9b). We found that a unanimous inferior clinical outcome of glioma patients of relatively higher risk value across all 3 cohorts, which were further support by an exceedingly satisfactory AUC value above 0.9 at 1 year and 3 years post-operative time points (Fig. 9c). PTN and CNN3 were found as 2 DE-ARGs with highest hazard ratio (Fig. 9d). We further integrated age into the nomogram model for better clinical practice of our prognostic model (Fig. 9e).

## Discussion

In our study, we aimed to characterize the gene expression patterns and interactions within the TME of glioma. We identified four distinct malignant cell subclusters and examined their specific gene expression profiles. Additionally, we focused on immune-related genes and identified 15 differentially expressed immune-related genes (DE-ARGs) that were specifically expressed in the malignant glioma cell populations. Further analysis revealed that glioma cells with higher expression of these DE-ARGs were associated with gliogenesis, glial cell development, and vasculature development. To understand the cellular interactions within the TME, we investigated the main interacting cell type in glioma and found tumor-infiltrating monocytes to be predominant. These findings suggest the importance of immune cell infiltration in the glioma microenvironment. Furthermore, by utilizing the expression patterns of the 15 screened DE-ARGs, we categorized glioma samples into two molecular clusters with distinct immune features. This observation suggests a potential relationship between angiogenesis and immune activation and recruitment in glioma. Finally, we developed a prognostic model based on the expression levels of the 15 DE-ARGs and evaluated its predictive ability for glioma patient outcomes. Our results demonstrated that this prognostic model exhibited high efficacy in predicting glioma patient outcomes.

We found CHI3L1 significantly down-regulated in the malignant glioma subclusters 1 and 3, which were identified as the preliminary states of malignant cell differentiation. Chitinase-3-like-1 protein, commonly referred to as YKL-40, is a glycoprotein belonging to the 18-glycosyl hydrolase family [11]. CHI3L1 has been implicated in various biological processes and diseases, including cancer [12]. However, its exact function and significance in different types of tumors are not fully understood. CHI3L1, a secreted protein, in glioblastoma and its association with radiation response and prognosis. A large-scale study of glioblastoma patients who underwent either subtotal resection or gross-total resection showed that higher expression of CHI3L1 was significantly associated with poorer radiation response, shorter time to progression, and shorter overall survival in the subtotal resection group. These associations were validated in the gross-total resection group. In multivariate analysis, CHI3L1 was found to be an independent predictor of survival, after adjusting for other factors. Additionally, CHI3L1 expression was significantly associated with genetic abnormalities in glioblastomas, specifically loss at chromosome 10q [13]. As regard to molecular mechanisms, CHI3L1 mRNA was found to be abundant in glioma cells and reactive astrocytes, while low in neurons and macrophages through in situ hybridization approaches. CHI3L1 expression was higher in GBMs compared to anaplastic oligodendrogliomas, and among GBMs, tumors with EGFR amplification or elevated EGFR expression had lower CHI3L1 expression [14]. In another study based on the bioinformatic analysis, low-grade glioma patients with low expression levels of EMP3 and CHI3L1 had a better prognosis. Furthermore, a correlation between EMP3 and CHI3L1 expression was found [15]. We provided a deeper insight into the expression of CHI3L1 in the glioma malignant cells by integrating scRNA-seq data into the analysis, which displayed differential expression pattern of CHI3L1 in glioma. These findings suggest that CHI3L1 is primarily produced by neoplastic glial cells and is associated with specific molecular alterations in gliomas. Further research is needed to fully understand the functional role and clinical implications of CHI3L1 in glioma progression and treatment response.

The main interacting cell type in the TME of glioma was found to be CAMs, while neutrophils had the lowest interaction intensity. The highest interaction was observed between intratumor monocytes and macrophages, fibroblasts, indicating an auto-secretory phenotype of macrophages and a para-secretory pattern between CAFs and CAMs. The TME, which consisted complex cellular and non-cellular components, plays a crucial role in cancer progression. The extracellular matrix (ECM) composition of cancer proves to be of grave importance in cancer progression [16]. In the context of GBM, the ECM composition undergoes significant changes, which contribute to the increased invasiveness and migration of glioma cells. Previous studies have identified several key components of the ECM, including hyaluronic acid (HA), fibronectin, thrombospondin, and tenascin-C, that are secreted by glioma cells and play a role in this process. Specifically, the increased expression of fibronectin and HA in the ECM, along with the upregulation of specific receptors and integrins on glioma cells, promote their mobility and invasiveness [17]. Glioma cells can enhance their expression of CD44, the primary surface receptor for HA, which also binds to matrix metalloproteinase 9 (MMP9) present in the ECM [18]. This interaction between CD44 and MMP9 facilitates glioma cell migration, which was also found to be highly enriched in mediating signal transduction in our study. The TME of GBM also undergoes physical changes, including edema and cellular compression, which act as physical stressors and increase the stiffness of the tumor. These physical alterations can further promote gliomagenesis. Interestingly, studies using murine models have revealed an immunomodulatory role of the tumor ECM. The ECM was found to be involved in CD47-mediated macrophage phagocytosis signaling through the expression of the tumor-associated extracellular matrix protein tenascin C (TNC). This interaction between TNC and macrophages highlights the intricate relationship between the innate immune system and the tumor ECM in GBM [19]. The production of periostin by glioma stem cells has been identified as a key factor in promoting extravasation and migration of peripheral monocytes and M2-like TAMs in the glioma environment [20]. These interactions are facilitated by the binding of periostin to αVβ3 integrins on the cell surface of monocytes and TAMs. Additionally, hypoxia can activate vascular endothelial growth factor receptor 1 (VEGFR1) and neuropilin-1 (NRP1) in monocytes, leading to their chemotaxis towards glioma and breast cancer cells [21]. The release of VEGFA and semaphorin 3A (Sema 3A) by tumor cells, including glioma, further activates NRP1, triggering the activation of VEGFR1 and subsequent recruitment of TAMs in glioma [22]. This complex interplay between glioma cells, periostin, monocytes, TAMs, and various signaling molecules highlights the importance of understanding the molecular mechanisms governing glioma progression and potential targets for therapeutic intervention. Therefore, further understanding of the complex interplay between glioma cells, the ECM, and CAMs is crucial for the development of targeted therapies for GBM.

Migrating ECs have the ability to overcome hypoxia and nutrient deprivation-induced ECs apoptosis and promote angiogenesis. This process is mediated by the action of VEGF and the adhesion of ECs to ECM molecules, which activate anti-apoptotic genes through the PI3K/Akt or NF-κB signaling pathways [23, 24]. In glioma, tumor cells release VEGF, which stimulates EC proliferation and angiogenesis. Interestingly, although TNF can induce EC apoptosis, it does not inhibit angiogenesis in glioma. This may be due to the activation of NF-κB in ECs, which allows them to evade TNF-induced apoptosis [25]. Experiments using a co-culture system of human brain microvascular endothelial cells (HBMVECs) and U251 glioma cells have shown that EC apoptosis induced by serum starvation can be reversed by recombinant VEGF protein and a culture medium from hypoxic glioma cells. Hypoxia treatment also activates TNF-induced VEGF and NF-κB signaling, leading to the upregulation of antiapoptotic genes, such as Bcl-2, Bcl-XL, survivin, and X-chromosome-linked inhibitor of apoptosis protein (XIAP), in ECs [26].

While our study provides significant insights into the gene expression patterns and interactions within the TME of gliomas, several limitations should be noted [27, 28]. First, our analysis relies heavily on bulk RNA-seq and scRNA-seq data from publicly available datasets, which may introduce inherent biases and limit the generalizability of our findings. Second, the identification and characterization of malignant cell subclusters were based on computational methods and would benefit from further experimental validation. Additionally, the prognostic model constructed using the 15 differentially expressed immune-related genes (DE-ARGs) requires validation in larger, independent cohorts to confirm its robustness and clinical utility. Lastly, while we identified key interactions within the TME, the complex dynamics and potential functional implications of these interactions were not fully explored, warranting further in-depth studies.

In summary, our study provides valuable insights into the gene expression patterns and cellular interactions within the TME of glioma. Additionally, our findings suggest a potential link between angiogenesis and immune activation in glioma, which may have important implications for therapeutic strategies and prognostic prediction in this disease.

## Conclusion

We characterized different malignant cell subclusters in glioma and investigate their gene expression patterns and interactions within TME. We constructed a prognostic model based on the expression levels of the 15 DE-ARGs and evaluated its predictive ability for glioma patient outcomes, which displayed exceedingly high efficacy.

## Data Availability

The data could be obtained from TCGA database,or coresponding author after the requestion.
